# Supplementary material for: Benefits and Harms of Treatment and Preventive Interventions for Hereditary Angioedema: Protocol for a Systematic Review and Network Meta-Analysis of Randomized Controlled Trials
Source: Genes (Basel). 2022 May 22;13(5):924. doi: 10.3390/genes13050924 (PMC9141233; doi:10.3390/genes13050924)
Supplement: Supplementary file 1 [file genes-13-00924-s001.zip › genes-1734095-supplementary.pdf]

Supplementary Online Material

Benefits and Harms of Treatment Interventions for Hereditary Angioedema: Protocol for a Systematic Review and Network Meta-Analysis of Randomized Controlled Trials

Authors: Mati Chuamanochan, Sutthinee Phuprasertsak, Fatima Itiritiphan, Chidchanok Ruengorn, Chabaphai Phosuya, Ratanaporn Awiphan, Brian Hutton, Kednapa Thavorn, Jonathan A Bernstein, Surapon Nochaiwong

Supplementary Online Content

**Table S1**	Systematic review search strategy	S3
**Table S2**	Grey literature from ongoing clinical trial register	S10

Table S1 Systematic review search strategy: MEDLINE (via OVID)

Search	Query	Items Found
#1	exp complement C1 inhibitor protein/	1226
#2	exp hereditary angioedema/	1248
#3	((hereditary or quincke or acquired) adj (angio$edema or $edema)).tw,kw,rn.	2360
#4	((C1 inhibitor or C1 INH) adj (deficiency or protein or HAE or angio$edema or $edema or AAE)).tw,kw,rn.	793
#5	(angio$edema or angioneurotic $edema or erythema marginatum or spontaneous edema attack* or asphyxia or HAE or HAE-FXII or HAE-PLG or HAE-ANGPTI or serine protease or SERPING1).tw,kw,rn	38900
#6	((skin or tongue or abdominal or digestive or gastrointestinal or airway or laryngeal) adj swell*).tw,kw,rn.	1007
#7	or/1-6	40175
#8	(therap* or treat* or manag* or on-demand or prophylaxis).mp	10324111
#9	(gene therap* or biologic* or monoclonal antibod* or kallikrein inhibitor* or antisense targeting prekallikrein or bradykinin b2 receptor antagonist* or C1 inhibitor* or plasma derived C1 inhibitor* or C1 esterase inhibitor* or factor XII inhibitor* or fresh frozen plasma or solvent detergent plasma or androgen* or hormone therap* or anti-fibrinolytic agent* or RNA interference targeted at FXII).tw,kw,rn	1305467
#10	((recombinant or human plasma or plasma derived or pasteurized or nanofiltered) adj3 (C1 inhibitor* or C1 INH or C1 esterase inhibitor*)).tw,kw,rn.	304
#11	(ecallantide or berotralstat or BCX7353 or ORLADEYO or KVD900 or kalbitor or icatibant or PHA022121 or firazyr or conestat alfa or ruconest or C1-INH or berinert or cinryze or haegarda or lanadelumab or ATN-249 or KDV824 or takhzyro or SHP643 or DX-2930 or avoralstat or garadacimab or PKK-Rx or IONIS-PKK or ALN-F12 or ARC-F12 or CSL312 or BMN331 or NTLA-2002 or danazol or stanozolol or oxandrolone or methyltestosterone or tibolone or oxymetholone or epsilon aminocaproic acid or EACA or tranexamic acid).tw,kw,rn	17521
#12	or/9-11	1318828
#13	7 and 8	15809
#14	7 and 12	5064
#15	or/13-14	17959
#16	(news or newspaper article or comment or editorial or interview or letter or review or systematic review or case report or case series or cross-sectional).pt.	5075729
#17	15 not 16	14323
#18	exp Clinical Trials as Topic/	360719
#19	randomi$ed controlled trial* or controlled clinical trial*	20131
#20	random allocation or double-blind method or single-blind method or clinical trial/	721378
#21	(controlled clinical trial) or (randomi$ed controlled trial) or (clinical trial).pt	565420
#22	(control* adj2 trial*).tw,kw.	304510
#23	((clinical adj trial*) or (randomly allocated) or (allocated adj2 random*) or randomi$ed or RCT$1 placebo*).tw,kw.	435124
#24	((singl* or doubl* or trebl* or tribl*) adj (blind* or mask* or dumm*)).tw,kw.	180636
#25	or/18-24	1456928
#26	17 and 25	986
#27	limit 26 to human	773

Table S1 Systematic review search strategy: Embase (via OVID) (Continued)

Search	Query	Items Found
#1	exp complement C1 inhibitor protein/	5089
#2	exp hereditary angioedema/	21327
#3	((hereditary or quincke or acquired) adj (angio$edema or $edema)).tw,kw,rn.	4468
#4	((C1 inhibitor or C1 INH) adj (deficiency or protein or HAE or angio$edema or $edema or AAE)).tw,kw,rn.	1458
#5	(angio$edema or angioneurotic $edema or erythema marginatum or spontaneous edema attack* or asphyxia or HAE or HAE-FXII or HAE-PLG or HAE-ANGPTI or serine protease or SERPING1).tw,kw,rn	56857
#6	((skin or tongue or abdominal or digestive or gastrointestinal or airway or laryngeal) adj swell*).tw,kw,rn.	1673
#7	or/1-6	69456
#8	(therap* or treat* or manag* or on-demand or prophylaxis).mp	13928711
#9	(gene therap* or biologic* or monoclonal antibod* or kallikrein inhibitor* or antisense targeting prekallikrein or bradykinin b2 receptor antagonist* or C1 inhibitor* or plasma derived C1 inhibitor* or C1 esterase inhibitor* or factor XII inhibitor* or fresh frozen plasma or solvent detergent plasma or androgen* or hormone therap* or anti-fibrinolytic agent* or RNA interference targeted at FXII).tw,kw,rn	1573202
#10	((recombinant or human plasma or plasma derived or pasteurized or nanofiltered) adj3 (C1 inhibitor* or C1 INH or C1 esterase inhibitor*)).tw,kw,rn.	638
#11	(ecallantide or berotralstat or BCX7353 or ORLADEYO or KVD900 or kalbitor or icatibant or PHA022121 or firazyr or conestat alfa or ruconest or C1-INH or berinert or cinryze or haegarda or lanadelumab or ATN-249 or KDV824 or takhzyro or SHP643 or DX-2930 or avoralstat or garadacimab or PKK-Rx or IONIS-PKK or ALN-F12 or ARC-F12 or CSL312 or BMN331 or NTLA-2002 or danazol or stanozolol or oxandrolone or methyltestosterone or tibolone or oxymetholone or epsilon aminocaproic acid or EACA or tranexamic acid).tw,kw,rn	37879
#12	or/9-11	1603251
#13	7 and 8	36611
#14	7 and 12	10650
#15	or/13-14	39942
#16	(news or newspaper article or comment or editorial or interview or letter or review or systematic review or case report or case series or cross-sectional).pt.	4557390
#17	15 not 16	31876
#18	exp Clinical Trials as Topic/	362216
#19	randomi$ed controlled trial* or controlled clinical trial*	500030
#20	random allocation or double-blind method or single-blind method or clinical trial/	998896
#21	(controlled clinical trial) or (randomi$ed controlled trial) or (clinical trial).pt	487489
#22	(control* adj2 trial*).tw,kw.	407561
#23	((clinical adj trial*) or (randomly allocated) or (allocated adj2 random*) or randomi$ed or RCT$1 placebo*).tw,kw.	630838
#24	((singl* or doubl* or trebl* or tribl*) adj (blind* or mask* or dumm*)).tw,kw.	244561
#25	or/18-24	2006020
#26	17 and 25	3606
#27	limit 26 to human	3395

Table S1 Systematic review search strategy: PubMed (Continued)

Search	Query	Items Found
#1	((complement c1 inhibitor protein[MeSH Terms]) OR (edema, hereditary angioneurotic[MeSH Terms])) OR (hereditary angioedema[MeSH Terms])	1787
#2	(((((((((((((((hereditary angioedema[Title/Abstract]) OR (quincke angioedema[Title/Abstract])) OR (acquired angioedema[Title/Abstract])) OR (C1 inhibitor deficiency[Title/Abstract])) OR (C1 INH[Title/Abstract])) OR (AAE[Title/Abstract])) OR (angioedema[Title/Abstract])) OR (angioneurotic edema[Title/Abstract])) OR (erythema marginatum[Title/Abstract])) OR (spontaneous edema attack*[Title/Abstract])) OR (asphyxia[Title/Abstract])) OR (HAE[Title/Abstract])) OR (HAE-FXII[Title/Abstract])) OR (HAE-PLG[Title/Abstract])) OR (serine protease[Title/Abstract])) OR (SERPING1[Title/Abstract])	40522
#3	#1 OR #2	40804
#4	((((therap*[Title/Abstract]) OR (treat*[Title/Abstract])) OR (manag*[Title/Abstract])) OR (on-demand[Title/Abstract])) OR (prophylaxis[Title/Abstract])	8243921
#5	(((((((((((((((gene therap*[Title/Abstract]) OR (biologic*[Title/Abstract])) OR (monoclonal antibod*[Title/Abstract])) OR (kallikrein inhibitor*[Title/Abstract])) OR (antisense targeting prekallikrein[Title/Abstract])) OR (bradykinin b2 receptor antagonist*[Title/Abstract])) OR (C1 inhibitor*[Title/Abstract])) OR (plasma derived C1 inhibitor*[Title/Abstract])) OR (C1 esterase inhibitor*[Title/Abstract])) OR (factor XII inhibitor*[Title/Abstract])) OR (fresh frozen plasma[Title/Abstract])) OR (solvent detergent plasma[Title/Abstract])) OR (androgen*[Title/Abstract])) OR (hormone therap*[Title/Abstract])) OR (anti-fibrinolytic agent*[Title/Abstract])) OR (RNA interference FXII[Title/Abstract])	1275046
#6	((recombinant C1 inhibitor*[Title/Abstract]) OR (plasma derived C1 inhibitor*[Title/Abstract])) OR (C1 inhibitor* pasteurized[Title/Abstract])) OR (C1 inhibitor* nanofiltered[Title/Abstract])) OR (C1 INH[Title/Abstract])) OR (C1 esterase inhibitor*[Title/Abstract])) OR (IgG1 monoclonal antibody inhibitor*[Title/Abstract])) OR (ecallantide[Title/Abstract])) OR (berotralstat[Title/Abstract])) OR (BCX7353[Title/Abstract])) OR (ORLADEYO[Title/Abstract])) OR (KVD900[Title/Abstract])) OR (kalbitor[Title/Abstract])) OR (icatibant[Title/Abstract])) OR (PHA022121[Title/Abstract])) OR (firazyr[Title/Abstract])) OR (conestat alfa[Title/Abstract])) OR (ruconest[Title/Abstract])) OR (berinert[Title/Abstract])) OR (cinryze[Title/Abstract])) OR (haegarda[Title/Abstract])) OR (lanadelumab[Title/Abstract])) OR (ATN-249[Title/Abstract])) OR (KDV824[Title/Abstract])) OR (takhzyro[Title/Abstract])) OR (SHP643[Title/Abstract])) OR (DX-2930[Title/Abstract])) OR (avoralstat[Title/Abstract])) OR (garadacimab[Title/Abstract])) OR (PKK-Rx[Title/Abstract])) OR (IONIS-PKK[Title/Abstract])) OR (ALN-F12[Title/Abstract])) OR (ARC-F12[Title/Abstract])) OR (CSL312[Title/Abstract])) OR (BMN331[Title/Abstract])) OR (NTLA-2002[Title/Abstract])) OR (danazol[Title/Abstract])) OR (stanozolol[Title/Abstract])) OR (oxandrolone[Title/Abstract])) OR (methyltestosterone[Title/Abstract])) OR (tibolone[Title/Abstract])) OR (oxymetholone[Title/Abstract])) OR (epsilon aminocaproic acid[Title/Abstract])) OR (EACA[Title/Abstract])) OR (tranexamic acid[Title/Abstract])	14844
#7	#5 OR #6	1285960
#8	#3 AND #4	13687
#9	#3 AND #7	5152

#10	#8 OR #9	16196
#11	(((((((Case Reports[Publication Type]) OR Comment[Publication Type]) OR Editorial[Publication Type]) OR Guideline[Publication Type]) OR Letter[Publication Type]) OR News[Publication Type]) OR Newspaper Article[Publication Type]) OR Review[Publication Type]	6861400
#12	#10 NOT #11	11419
#13	Filters applied: Clinical Study, Clinical Trial, Clinical Trial, Phase II, Clinical Trial, Phase III, Clinical Trial, Phase IV, Comparative Study, Controlled Clinical Trial, Pragmatic Clinical Trial, Randomized Controlled Trial, Humans.	1117

Table S1 Systematic review search strategy: Cochrane Library (Continued)

Search	Query	Items Found
#1	MeSH descriptor: [Complement C1 Inactivator Proteins] explode all trees	117
#2	MeSH descriptor: [Angioedemas, Hereditary] explode all trees	120
#3	C1 INH OR hereditary angioneurotic edema OR quincke angioedema OR acquired angioedema OR C1 inhibitor deficiency	329
#4	#1 OR #2 OR #3	436
#5	therap* OR treat* OR manag* OR on-demand OR prophylaxis	1179276
#6	gene therap* OR biologic* OR monoclonal antibod* OR kallikrein inhibitor* OR antisense targeting prekallikrein OR bradykinin b2 receptor antagonist* OR C1 inhibitor* OR plasma derived C1 inhibitor* OR C1 esterase inhibitor* OR factor XII inhibitor* OR fresh frozen plasma OR solvent detergent plasma OR androgen* OR hormone therap* OR anti-fibrinolytic agent* OR RNA interference FXII	113394
#7	recombinant C1 inhibitor* OR plasma derived C1 inhibitor* OR C1 inhibitor* pasteurized OR C1 inhibitor* nanofiltered OR C1 esterase inhibitor* OR IgG1 monoclonal antibody inhibitor* OR ecallantide OR berotralstat OR BCX7353 OR ORLADEYO OR KVD900 OR kalbitor OR icatibant OR PHA022121 OR firazyr OR conestat alfa OR ruconest OR berinert OR cinryze OR haegarda OR lanadelumab OR ATN-249 OR KDV824 OR takhzyro OR SHP643 OR DX-2930 OR avoralstat OR garadacimab OR PKK-Rx OR IONIS-PKK OR ALN-F12 OR ARC-F12 OR CSL312 OR BMN331 OR NTLA-2002 OR danazol OR stanozolol OR oxandrolone OR methyltestosterone OR tibolone OR oxymetholone OR epsilon aminocaproic acid OR EACA OR tranexamic acid	5676
#8	#6 OR #7	117423
#9	#4 AND #5	407
#10	#4 AND #8	416
#11	#9 OR #10	435
#12	Limit to Trials	416

Table S1 Systematic review search strategy: Scopus (Continued)

Search	Query	Items Found
#1	TITLE-ABS-KEY (“hereditary angioedema” OR “complement c1 inhibitor protein” OR “C1 INH” OR “hereditary angioneurotic edema” OR “quincke angioedema” OR “acquired angioedema” OR “C1 inhibitor deficiency”)	3845
#2	TITLE-ABS-KEY (therap* OR treat* OR manag* OR on-demand OR prophylaxis)	16862474
#3	#1 AND #2	2408
#4	TITLE-ABS-KEY (“gene therap*” OR biologic* OR “monoclonal antibod*” OR “kallikrein inhibitor*” OR “antisense targeting prekallikrein” OR “bradykinin b2 receptor antagonist*” OR “C1 inhibitor*” OR “plasma derived C1 inhibitor*” OR “C1 esterase inhibitor*” OR “factor XII inhibitor*” OR “fresh frozen plasma” OR “solvent detergent plasma” OR androgen* OR “hormone therap*” OR “anti-fibrinolytic agent*” OR “RNA interference FXII”)	3417081
#5	TITLE-ABS-KEY (“recombinant C1 inhibitor*” OR “plasma derived C1 inhibitor*” OR “C1 inhibitor* pasteurized” OR “C1 inhibitor* nanofiltered” OR “C1 esterase inhibitor*” OR “IgG1 monoclonal antibody inhibitor*” OR ecallantide OR berotralstat OR BCX7353 OR ORLADEYO OR KVD900 OR kalbitor OR icatibant OR PHA022121 OR firazyr OR “conestat alfa” OR ruconest OR berinert OR cinryze OR haegarda OR lanadelumab OR ATN-249 OR KDV824 OR takhzyro OR SHP643 OR DX-2930 OR avoralstat OR garadacimab OR PKK-Rx OR IONIS-PKK OR ALN-F12 OR ARC-F12 OR CSL312 OR BMN331 OR NTLA-2002 OR danazol OR stanozolol OR oxandrolone OR methyltestosterone OR tibolone OR oxymetholone OR epsilon aminocaproic acid OR EACA OR tranexamic acid)	2684
#6	#4 OR #5	3419362
#7	#1 AND #6	2808
#8	#3 OR #7	3327
#9	Filters: (EXCLUDE (DOCTYPE , "re") OR EXCLUDE (DOCTYPE , "le") OR EXCLUDE (DOCTYPE , "no") OR EXCLUDE (DOCTYPE , "sh") OR EXCLUDE (DOCTYPE , "ed") OR EXCLUDE (DOCTYPE , "ch") OR EXCLUDE (DOCTYPE , "er") OR EXCLUDE (DOCTYPE , "bk") OR EXCLUDE (DOCTYPE , "cr") OR EXCLUDE (DOCTYPE , "tb"))	2515

Table S1 Systematic review search strategy: CINAHL (Continued)

Search	Query	Items Found
#1	AB (“hereditary angioedema” OR “complement c1 inhibitor protein” OR “C1 INH” OR “hereditary angioneurotic edema” OR “quincke angioedema” OR “acquired angioedema” OR “C1 inhibitor deficiency”)	343
#2	AB (therap* OR treat* OR manag* OR on-demand OR prophylaxis)	1468772
#3	S1 AND S2	287
#4	AB (“gene therap*” OR biologic* OR “monoclonal antibod*” OR “kallikrein inhibitor*” OR “antisense targeting prekallikrein” OR “bradykinin b2 receptor antagonist*” OR “C1 inhibitor*” OR “plasma derived C1 inhibitor*” OR “C1 esterase inhibitor*” OR “factor XII inhibitor*” OR “fresh frozen plasma” OR “solvent detergent plasma” OR androgen* OR “hormone therap*” OR “anti-fibrinolytic agent*” OR “RNA interference FXII”)	85911
#5	AB (“recombinant C1 inhibitor*” OR “plasma derived C1 inhibitor*” OR “C1 inhibitor* pasteurized” OR “C1 inhibitor* nanofiltered” OR “C1 esterase inhibitor*” OR “IgG1 monoclonal antibody inhibitor*” OR ecallantide OR berotralstat OR BCX7353 OR ORLADEYO OR KVD900 OR kalbitor OR icatibant OR PHA022121 OR firazyr OR “conestat alfa” OR ruconest OR berinert OR cinryze OR haegarda OR lanadelumab OR ATN-249 OR KDV824 OR takhzyro OR SHP643 OR DX-2930 OR avoralstat OR garadacimab OR PKK-Rx OR IONIS-PKK OR ALN-F12 OR ARC-F12 OR CSL312 OR BMN331 OR NTLA-2002 OR danazol OR stanozolol OR oxandrolone OR methyltestosterone OR tibolone OR oxymetholone OR epsilon aminocaproic acid OR EACA OR tranexamic acid)	2216
#6	S4 OR S5	87745
#7	S1 AND S6	245
#8	S3 OR S7: Expanders Apply equivalent subjects	311

Table S2 Grey literature from ongoing clinical trial register

Ongoing clinical trial register

- Australia and New Zealand's (ANZCTR) (http://www.anzctr.org.au)
- Brazilian Clinical Trials Registry (ReBec) (http://www.ensaiosclinicos.gov.br)
- Chinese Clinical Trial Registry (ChiCTR) (http://www.chictr.org.cn)
- Clinical Research Information Service (CRiS), Republic of Korea (http://cris.cdc.go.kr)
- Clinical Trials Registry - India (CTRI) (http://ctri.nic.in)
- Cuban Public Registry of Clinical Trials(RPCEC) (http://registroclinico.sld.cu)
- EU Clinical Trials Register (EU-CTR) (https://www.clinicaltrialsregister.eu)
- German Clinical Trials Register (DRKS) (http://www.drks.de)
- Iranian Registry of Clinical Trials (IRCT) (http://www.irct.ir)
- Japan Primary Registries Network (https://rctportal.niph.go.jp)
- The Netherlands Trial Register (http://www.trialregister.nl)
- Pan African Clinical Trial Registry (PACTR) (http://www.pactr.org)
- Peruvian Registry of Clinical Trials (http://www.ins.gob.pe/ensayosclinicos)
- Philippine Health Research Registry (http://registry.healthresearch.ph)
- Sri Lanka Clinical Trials Registry (SLCTR) (http://www.slctr.lk)
- South African National Clinical Trials Register (http://www.sanctr.gov.za)
- Swiss FOPH Human Research Projects (https://www.kofam.ch/en/swiss-clinical-trials-portal.html)
- Tanzania Clinical Trial Registry (http://www.tzctr.or.tz)
- Thai Clinical Trials Registry (http://www.clinicaltrials.in.th)
- The United Kingdoms' ISRCTN registry (http://www.isrctn.com)
- The US National Institutes of Health Ongoing Trials Registry (http://clinicaltrials.gov)
- The World Health Organization International Clinical Trials Registry Platform (ICTRP) (https://www.who.int/ictrp)

Preprint databases

- medRxiv (https://www.medrxiv.org)
- bioRxiv (https://www.biorxiv.org)
- Research Square (https://www.researchsquare.com)
